# Buffalo Academy of Medicine

**Published:** 1901-02

**Authors:** Julius Ullman


### BUFFALO ACADEMY OF MEDICINE.

*Section on Medicine, December 10, 1900.*

Reported by JULIUS ULLMAN, M. D., Secretary.

THE third regular meeting for the season of the medical section of the Buffalo Academy of Medicine was held in the Public Library Building, Tuesday evening, December 10, 1900.

The chairman, Dr. Eli H. Long called the section to order and the minutes of the previous meeting were read and approved.

It was moved by Dr. A. L. Benedict and seconded by Dr. Charles S. Jewett that in order to shorten the duration of meetings the formal minutes be limited to a brief record of the transactions of the session, but that this be not construed as an objection to the publication of the reports. Lost.

Dr. Prescott LeBreton presented a paper which in the unavoidable absence of the essayist, was read by Dr. Julius Ullman, entitled,

### THE TREATMENT OF DISEASE LOCALLY BY HOT AIR.

The writer referred to Tallerman, of England, who in 1893, introduced the use of superheated dry air as a remedy to alleviate pain and cure disease. A description of the apparatus, which essentially consists of (1) an oven to enclose the affected part (2) a suitable covering for the skin to absorb perspiration and prevent blistering and (3) some means of generating heat.

The treatment to any given part occupies from one half to one hour and the temperature reached in the bath varies from 250° to 340° F., the degree of heat required depending upon the sensation of the patient. Massage is indicated afterward, especially if there is any swelling or exudation. The effects of this extreme heat locally are: a relaxation of the tissues, an increase in the circulation with dilatation of the bloodvessels, a numbing of the sensory nerves, a marked perspiration and often a change from an unhealthy to a healthy metabolism.

Swelling is diminished, pain is relieved, stiffened joints and muscle-nerves move freely.

The writer detailed the effect of this treatment in twelve selected cases, including Colles's fracture, sciatica, lithemic pains, contusion to lumbar region, coccygodynia rheumatoid arthritis, neurasthenia spinalis, old dislocation of the hip, subacute rheumatism and neuritis, in all of which good results were noted with oftentimes a cessation of all symptoms and cure. In some cases but one treatment was required, in others many were given. A similar result was also noted in thirty-five cases selected from the literature of the subject.

DISCUSSION.

Dr. Irving M. Snow heartily commended this form of treatment, and said it is superior in rheumatoid arthritis. He had arthritis of the shoulder joint of six months' duration, which was cured by this form of treatment. The heat was applied for half hour, after which Swedish movements were given.

Dr. Fridoln Thoma, stated that he has used this method of treatment in muscular rheumatism and neuritis, but does not believe that he used such a high degree of temperature as is stated in the paper. He asked whether such a high degree of temperature could alter the circulating bloodcells.

[Note.—Dr. Ullman calls attention to the leucocytosis as occurring after cold baths and massage. Winternitz and Thayer have shown a leucocytosis following the cold bath in typhoid fever. The general stimulation of heat or cold and the mechanical effect of massage would produce this effect, by causing a return to the circulation of leucocytes stagnant in various parts of the body. The effect of heat generally applied for a sufficient length of time will induce sweating, which if in excess would produce a similar result as severe diarrheas, such as in cholera—namely, an inspissation of blood. In this condition the solid elements of the blood increase relatively as the fluid portion decreases. In accordance with the inspissation the red and white corpuscles increase per cubic millimeter, and so also the specific gravity.]

Dr. Charles G. Stockton read a paper on

### CYCLIC OR PERIODIC VOMITING IN CHILDHOOD.

The writer reported a case of what was held to be characteristic cyclic or periodic vomiting in a child. It first appeared when the child was three years old, and occurred at intervals of six or twelve weeks thereafter, for a period of five years, when the attacks declined in frequency and severity, and have now quite disappeared. The seizures were ushered in by a day or two of dulness with inactivity of the bowels; the appetite usually good. The vomiting occurred suddenly and was repeated sometimes with severe retching at intervals of half an hour or an hour. The ejecta appeared to be pure gastric juice, rich in hydrochloric acid, which acidity declined as the attacks passed off. After vomiting for about a week, during which there was very alarming prostration, constipation of the bowels and diminution of the urinary output, improvement appeared as suddenly as did the onset. In forty-eight hours the child seemed as well as ever, and continued to be perfectly well in the interval. The affection was apparently in no way related to any other ascertainable condition, and was not precipitated by over-eating or other faults in hygiene. The writer assigned this case to a group which he thinks differs from the cases reported by Leyden, which occurred in adults, and those reported by Boas, in which there was hypochlorhydria, and from other groups not answering to the description of pure cyclic vomiting in children. Hypodermoclysis and enteroclysis were recommended as means of averting the dangers of collapse. Several fatal cases were referred to.

### DISCUSSION.

Dr. Irving M. Snow referred to his case of cyclic or periodic vomiting noted in Dr. Stockton's paper. In this case the history pointed strongly to a neurosis. The father of the child was an alcoholic and died in an insane asylum; his father was alcoholic; the child's mother had convulsions; four other children had convulsions, and one died of epilepsy. The child was nineteen months old when first attacked and when seen was eight years old. The vomiting came on periodically and was then continuous, so that such an attack gave one the impression of a tubercular meningitis, *i. e.*, vomiting, irregular pulse and respiration, scathiod abdomen, and the like. The bowels were regular and there was no fecal accumulation and therefore no intestinal toxemia. The symptoms ameliorated as the child grew older and treatment had but little effect. Dr. Snow asked whether an excess of hydrochloric acid was noted in Dr. Stockton's case.

Dr. Dewitt Sherman said that we should not overlook the foods ingested preceding an attack. He referred to gastric cases of vomiting occurring after children's tea parties and following exposure to cold. Dr. Sherman saw a case this summer which was also seen by Dr. Holt, of New York, in which a child, after taking a cold bath and then partaking of chicken, which was afterwards found to have been preserved in cold storage was attacked with severe vomiting and prostration, and the urine showed albumen and casts. This was called by Holt cyclic vomiting. A peculiar phenomenon noted in the child's case was a reversed peristalsis of the small intestine. It seemed that all those who partook of this chicken were attacked with vomiting and diarrhea and Dr. Sherman therefore differs with the diagnosis of Dr. Holt and regards this case as one of ptomaine poisoning. The fact that there was albuminuria and casts suggests that this was more than a neurosis. This is also seen in the autopsy of one of Griffith's cases in which there was a necrotic area found in the walls of the stomach. Dr. Sherman's case afterward developed pneumonia and later a basilar meningitis, but there was no tubercular history in the case.

Dr. DeLancey Rochester said that the case mentioned by Dr. Sherman was evidently one of ptomaine poisoning, and should not be classed with the case reported by Dr. Stockton. Dr. Stockton's case belongs to a class occurring in children who are well in all other respects in whom the attacks of vomiting come on at regular intervals with languor and constipation.

Dr. Eli H. Long reported a case of periodic vomiting in a child five years old. The doctor saw the case first in November, 1899. At this time the attacks came on every month, lasting about a week. There was no fever. The child died in the last attack, which was the sixth seen by the doctor.

Dr. Chas. G. Stockton asserted that these cases when first seen do resemble a tubercular meningitis, but the history of previous attacks is of value. In answer to Dr. Snow he said that in his case the excess of hydrochloric acid secretion was noted as part of the affection. The stomach contents were examined in 20-25 attacks and were found to consist of pure gastric juice with an excess of hydrochloric acid. As the attacks subsided the hydrochloric acid was diminished. The hyperchlorhydria is then a part of the disease, unless there exists a primary diseased condition which will not allow its secretion. Dr. Sherman's case can be classified only after additional data, but it evidently is not one of cyclic vomiting. As to the etiology, like migraine and other allied affections, it seems an easy way of disposing of the case to call it a neurosis, but this does not exclude some possible form of infection.

Ratchford claims an increase in uric acid to be a cause. Forscheimer speaks of a dilatation of the colon. Constipation is noted during the attack, but indican is not found until the last. Acetone and casts were found in Griffith's case. The theory of necrosis as noted in Griffith's case may be the same as that noted in cholera, due to the great starvation of the tissues for water, and Johnston, of Washington, therefore recommends enteroclysis and hypodermoclysis.

Attendance, 30.

---

*Section on Ophthalmology, Otology, Rhinology and Laryngology, December 17, 1900.*

Reported by GEORGE F. COTT, M. D., Secretary.

The fourth meeting of this section for the season was held December 17, 1900, Dr. Frank Whitehill Hinkel in the chair.

Dr. J. C. Clemesha described a case with a foreign body in the lens. In the last week of November, according to the patient, while working at the locomotive shops at Depew, something flew in his left eye. He was not incapacitated, however, and continued the work until the second week in December, when he visited the Buffalo Eye and Ear Infirmary.

On examination of the eye, vision was 20-70; there was no congestion, pain or photophobia. With focal illumination there showed on the anterior capsule of the lens, and in its upper and inner quadrant an irregular shaped opacity, gray in color, and not exceeding .5 centimeter in any direction. In the center of this opacity was a minute dark spot and behind and in the substance of the lens was another minute opaque spot somewhat triangular in shape. The rest of the lens-capsule was clear. The condition pointed to foreign body lodged in the lens substance, the dark spot in the capsular opacity being the point of entrance, while the corneal had cleared in a like manner as after a needling operation. The nature of the substance, metallic or otherwise, could only be determined by the use of the sidereoscope.

In discussing the case described by Dr. Clemesha, Dr. Hubbell said that, although there was no scar or other evidence of a wound of the cornea, yet the opaque dot on the anterior capsule of the lens with a black-looking speck at its center, and a further opacity in the lens substance near its center, which showed a doubtful reflex of the ophthalmoscope, would lead him to suspect the presence in the lens of a minute foreign body. Moreover, the patient believed the vision was affected in that eye since the time of the supposed injury.

Dr. A. A. HUBBELL then read a paper entitled,

THE ETIOLOGY AND TREATMENT OF CONCOMITANT STRABISMUS—A RECONSIDERATION.

After referring to the histories of the theories of strabismus, he discussed, briefly, the physiology of vision, and then detailed the cause of strabismus. He drew the conclusion that it was primarily and essentially a nerveopathic condition, and entirely discarded the theory that it is muscular, as is so often held. There might be secondary changes affecting the ocular muscles and adjoining aponeurotic tissues, but these were not primary. The child had normal muscles before squinting, as shown by the normal ocular movements. Squint, when present, disappears under deep narcosis. Dr. Hubbell said the rotation of squinting eyes, in which secondary changes have not taken place, are of full normal extent.

The treatment, even operative, had reference only to the changes of the forces of innervation. The so-called functional or optical treatment will be successful in the majority of cases, if it can be commenced early enough. In convergent strabismus, which begins at one and a half to three and a half years of age, glasses would restore parallelism, if worn constantly, as soon as child would be old enough to do so without accident. After squint has existed permanently for three to five years, the spectacle treatment will be successful only in a small portion of cases. After glasses have been tried several months or a year without success, a properly selected operation may then be resorted to.

DISCUSSION.

Dr. GEORGE C. BLACKHAM, of Dunkirk, said he came to learn rather than to express an opinion. He intended to touch upon only one point—the general physiology. The human mind seeks some simple explanation for all cases. That is not good physiology. It is quite as possible to be the external muscle of the eye, which is deficient, as the other. We don't know why some parts of the body should be stronger, longer, or weaker than other parts. So in many cases we see, simple causes are quite possible. Many causes contribute to the same result.

Dr. Lucien Howe said he could see no reason for prescribing convex glasses for children who are hardly more than infants. A glass itself is a dangerous neighbor to the eye under such circumstances, and it is possible to obtain relaxation of t..e ciliary muscle more completely by weak solutions of belladonna. It is well-established that this can be used constantly for months or even years without any evil effects and in these young children he has frequently observed a convergent strabismus disappear when only occasional applications were made. Indeed, it is a question if this would not occur in a considerable percentage without any treatment whatever. In all cases, however, the children would be taught a few letters at as early an age as possible, in order that the vision of each eye might be accurately tested, and if this begins to decrease then, and not until then, more active treatment or operation is advisable.

Dr. A. G. Bennett then read a paper on

THE INFLUENCE OF THE VERTICAL MUSCLES IN THE PRODUCTION OF ASTHENOPIA.

He said: the most fascinating work of the ophthalmologist is, in my opinion, the correction of muscular anomalies. It is to the initiated a simple matter to make a diagnosis of heterophoria, and to correct with a prism or a tenotomy the offending muscles, but an experience of nine years taught me that nothing is so elusive and no condition of the eyes so full of seeming contradictions as a case of heterophoria. When first I undertook this work a case of esophoria was treated by tenotomy of the internal rectus or by a prism base out, esophoria by tenotomy of the internal or prism base in, hyperphoria by prism base down or tenotomy of superior rectus. The question of latent errors was much insisted on and a patient was given prisms to develop or make manifest the supposed hidden error. A more extensive experience has shown me that a prism of tenotomy of either of the lateral muscles seldom is of much avail in correcting a horizon deviation.

If the prisms be used, they act only as a crutch to a weak muscle, which, by lack of use, becomes weaker still, or if operation be done, sooner or later, the old trouble is back with all its virulence. With the exception of a full tenotomy to correct a convergent or divergent squint, and which I have to plead guilty to having performed several times with a mental resolve that, God being willing, one of these days sufficient knowledge will be vouchsafed me that I may relegate to the limbs of obsolete operations this unscientific procedure, I am proud to say, I have not operated on an internal rectus for esophoria or on an external rectus for exophoria for the last five years. Nor have I prescribed prisms more than three or four times for either condition, or have they been successful when prescribed.

From an experience garnered from some 3,000 private cases and 1,500 dispensary patients during the last five years, not all to be sure cases of muscular trouble, but having among them a considerable number, I have arrived at some conclusions which are satisfactory to myself, which have enabled me to relieve my patients, and I trust not without some interest to other members of the profession.

To begin with esophoria, Dr. Gould, some years ago when editor of the *Medical News* of Philadelphia, expressed his scepticism as to its frequent occurrence, saying that in an experience of some thousands of cases, it was the rarest condition. This is an expression of opinion that I can endorse, and without going into statistics, would state that 6° of esophoria is uncommon and then is accompanied by a hyperopia which, when corrected, almost invariably corrects the esophoria. If however this does not occur and symptoms of eyestrain are not relieved, look elsewhere for the trouble, and the most likely place to find it is in the superior or inferior rectus. The slightest vertical deviation may be the key to the whole problem. Latent esophoria or exophoria, I do not believe in, but latent hyperphoria I know exists; it can be demonstrated. I have seen only $\frac{1}{8}$° of hyperphoria, a difference in sursumduction of 3°. If now with painstaking care you arrive at the full amount of hyperphoria and correct preferably by tenotomy, no further trouble will be experienced from the esophoria.

Esophoria is apt to be more troublesome and run into higher degrees, but many cases of exophoria are dependent upon incorrected hyperphoria or anophoria, and simple cases that are not relieved by prism exercise will not get any permanent improvement from tenotomy of the external rectus, and that those cases that are relieved by a tenotomy would have done just as well by exercise. There still remains, however a class of cases in which none of the foregoing methods accomplish any good, and in these cases probably the error will be found in the declination of the vertical meridian and the adjustment of the eye to the correct position by the methods devised by Stevens, may prove the solution of this problem. This method, however, is too new for me to speak on with any feeling of assurance, though I have seen some good results at Dr. Stevens's hands, but have had no personal experience. In cases of marked hyper-exophoria, the correction of the hyperphoria and the establishment of strong convergent power, by means of prism exercise, has rendered me excellent servi ce; indeed, the correction of the hyperphoria alone, in many instances, has satisfied the patient though the exophoria was as much in evidence as ever.

The correction of hyperphoria is not a simple matter. The question at once arises, which eye is at fault? Is the hyperphoric eye in correct position, or is it too high, or is the other eye too low? a question that is not always easily solved. Or, again, are both eyes too high or too low? Here comes in the exceeding value of the tropometer,an instrument without which no ophthalmologist can keep house. It is a grevious mistake to cut a left superior rectus when the right eye is low and a prism base down before the left will not give your patient comfort when he needs a prism base up before the right; but the phorometer and Maddox rod will not give you the slightest inkling as to which muscle is at fault. The tropometer, too, is of great service in that class of cases which when perfectly refracted and showing orthophoria still manifest symptoms of eye-strain. Some of the happiest results I ever had have occurred in just this class of patients. The tropometer revealed anophoria and a tenotomy of both superior recti has proved the diagnosis correct by almost immediate relief from suffering.

Valuable as the tropometer is, it is sometimes difficult to determine in cases of hyperphoria which is the muscle at fault. The markings on the instrument are 10° apart and in cases in which the error is not large the movements of the eye both up and down may appear normal. In such cases I am in the habit of ordering a temporary prism base down before the hyperphoric eye, and in most cases this proves the correct treatment and a permanent prism or tenotomy of the offending superior rectus is performed. I have had some cases in which the prism in this position has not proved satisfactory and reversing the lens and putting it before the other eye has brought about the desired relief. Particularly true is this in elderly people and my experience is that although in the vast majority of vertical tensions the error will be either that of hyperphoria or anophoria, the majority of the minority will be found to be patients over fifty years of age.

There are cases of strabismus, either convergent or divergent, in which the relief of the vertical tension will do more for the cure of strabismus than a tenotomy of the internal or external rectus, and no case should be operated on until the upward and downward rotation has been thoroughly investigated. I showed a case of cured divergent strabismus before the surgical section of the Academy last April, in which the externi were intact and orthophoria was present with an adduction of 50°. My treatment in this case had been the dropping of the left eye 9° and the right 4°, which left the eyes level and straight. Exercises were given with prism base out until the weakened interni recovered their lost tone, when the patient was relieved of distressing headaches and a disfiguring ocular defect.

DISCUSSION.

Dr. LUCIEN HOWE.—The author has said in his discussion that he determined the exact position of the eye by means of the tropometer. I am quite sure, however, that a longer experience with that instrument would have shown that it is not possible to speak accurately of lowering one eye a certain number of degrees and elevating another a corresponding distance. For at least two years, I have made frequent use of this instrument, not only in private practice but also occasionally at the Buffalo Eye and Ear Infirmary. The fact is that if a patient is told to look up as far as he can and the degree of supraducation measured, the patient may in the next minute turn the eye still farther up, by five or even ten degrees. In other words, we can only approximate a measurement in this way by taking the average of a considerable number of observations. At no one time can we say the eye turns up six degrees or seven degrees or any other definite distance.

---

*Section on Pathology, December 18, 1900.*

REPORTED BY F. W. McGUIRE, M. D., Secretary.

The fourth regular meeting was held at the Academy rooms, Tuesday, December 18, 1900, twenty Fellows being present, with Dr. EUGENE A. SMITH in the chair, who called the section to order at 9 p.m.

It was ordered that the minutes for the meeting on October 16th be omitted; whereupon the minutes of the November meeting were read and approved.

Dr. EUGENE WASDIN, U.S. Marine Hospital Service, read a paper entitled,

THE ACUTE INFECTIOUS DISEASES.

The author prefaced his remaks by saying that the conclusions presented had been reached after a very careful study of the acute infections, more especially yellow fever, to which he had given close study, both clinically and experimentally.

The acute infections bear a very decided relationship to each other, including in the list Asiatic cholera. Peste, diphtheria, tuberculosis, typhus fever, typhoid fever, yellow fever, scarlet fever, whooping cough, measles, influenza, pneumonia and idiopathic tetanus. They embrace a large proportion of the diseases incident to man. He next discussed the three potentials of germ life: (1) the law of specificity, that each causative germ can produce but one disease; (2) that of producing toxins; (3) that of producing sepsis.

He went quite minutely into the question of natural immunity and took up the different theories relating thereto: (1) retention theory; (2) the abstraction theory; (3) phagocytosis; (4) alexin theory of Buchner; (5) antitoxin theory of Erlick. He showed that in each of these theories, with one exception of Buchner's, referred to a conferred immunity after or during the infection. He maintained that where there is natural immunity, in the true sense of the word, there is no initial intoxication or no disease. He showed how the growth of the germ was dependent upon environment, and cited yellow fever as an instance. The germ at a temperature of 30° to 40° F. will not produce the disease. A true primary immunity does exist in part, he thinks, to the alexins and in part to other conditions not fully known. For example, influence of atmospheric environment, as in case of scarlet fever.

The author next considered the different types of disease depending upon the development of its different potentials. If toxic, to a moderate degree there was produced the ephemeral type; if toxic to excess, the siderante type, while if the septic potential was predominate, you had the septic forms of disease. As a type of toxic diseases he took diphtheria and septic peste.

He then took up each disease and showed how this formula worked in each individuald isease. For example, yellow fever—there is a form of disease lasting but a few days, with slight symptoms, with slight intoxication, this would be the ephemeral type. Then again, you have the intensely toxic form, ending in death in forty-eight hours—the siderante type.

Lastly, you have the usual type, with its well defined clinical and pathological characteristics, the septic form of the disease.

Dr. Wasdin maintained that in yellow fever the mode of invasion was constantly by way of the respiratory tract. In this way he took up peste, typhoid fever, cholera, pneumonia, diphtheria, influenza, tetanus and tuberculosis, and showed how they each followed the above formula. He went into the mode of infection in peste quite carefully. He maintained that the explanation of Calamette regarding pneumonical peste as being secondary to a primary lymph infection was far fetched, and believes that this infection is primary in the lung.

In this connection he discussed origin of typhoid fever. He maintained whlle it was not impossible or improbable to have its origin in the intestinal tract, yet evidence was gradually accumulating to show that the disease is frequently a respiratory one: (1) In certain cases of typhoid the organism could not be found in the intestine and colonies were found in the lung; (2) The bacillus is not in the stools until a variable time after symptoms of infection appear; (3) the fact that the intestinal canal is so well protected with its tough lining, gastric juice, etc., compared to the frail tissues of the lung.

From the fact that many and probably all the infectious diseases were able to colonise in the lung and produce there specific disease, he reasoned that the water from our streets, lawns, and the like, should receive as much attention, as far as the protection against spread of the acute infections, as the drinking water.

In this relation he also urged more rigid inforcement of regulations looking to the abatement of the "spitting nuisance"; moreover, that all sputum from cases of acute infectious diseases should be sterilised with almost as much caution as the alvine discharges in cholera and typhoid fever.

DISCUSSION.

Dr. CHARLES G. STOCKTON had listened to the paper with keen interest. He could not understand why the author was so emphatic that infection took place through the lungs and not through other sources; he said he hesitates to accept that the law applies similarly in different diseases, and thought that there would be considerable change in the law before it could be accepted clinically.

Dr. DELANCEY ROCHESTER thought some of the operators might give us some light in the discussion of acute infection. He thought that in mild cases of infections we might have the formation of an antitoxin.

Dr. H. R. Gaylord did not see how tuberculosis could be applied to the law and did not think the intestinal tract should be left out of a consideration regarding the infection taken place there, because the germ was not found in the tract. He said the lungs were undoubtedly more easily infected than intestinal tract. He believes as Dr. Stockton that there would have to be more investigations before the law could be accepted clinically.

Dr. Ludwig Schroeter asked Dr. Wasdin to explain why the infection did not always give reactory symptoms at the point of infection.

Dr. Wasdin, in answer to Dr. Schroeter's question said he could not tell why, but cited twenty-five cases of typhoid in the Marine ward in which in every case there was a diseased area of lung. He said he thought foci were always present in lung tissue, but required very close examination to find it.

Dr. Charles G. Stockton wished to know why he considers the lung foci primary and not secondary.

Dr. Wasdin said he could not prove it to be so, but thought it was so, and hoped future investigations would prove it.

Dr. S. Y. Howell wished to know if the typhoid germ was found in lung before being found in the intestinal tract.

Dr. Wasdin said at the Pasteur Institute they found the greater infection about the second week, commencing very small on first day and increasing each day.

It was moved and seconded that a vote of thanks be extended to Dr. Wasdin for his interesting and instructive presentation of the subject. Carried.

Dr. A. L. Benedict gave some notes on Globulin and albumose as found in urine, with the view of soliciting coöperation in this work.

Discussion by Dr. Clowes.

---

Caution Regarding Herpes.—In the treatment of the various forms of herpes, it should be borne in mind that poultices, fomentations, lotions, and the like, are apt to soften the walls of the vesicles and facilitate their rupture, thereby prolonging instead of curtailing the disease, and adding considerable to the patient's discomfort. Powdered oleate of zinc is the best local application, of a weak solution of acetate of lead with glycerine.—*Jour. Med. and Science.*

